# Bond breakage under pressure in a metal organic framework†
†Electronic supplementary information (ESI) available: PXRD, SEM, FT-IR, fitting results of *R*-space EXAFS of UiO-66 nanocrystals. See DOI: 10.1039/c7sc03786d


**DOI:** 10.1039/c7sc03786d

**Published:** 2017-10-09

**Authors:** Zhi Su, Yu-Run Miao, Guanghui Zhang, Jeffrey T. Miller, Kenneth S. Suslick

**Affiliations:** a Department of Chemistry , University of Illinois at Urbana-Champaign , Urbana , Illinois 61801 , USA . Email: ksuslick@illinois.edu; b School of Chemistry and Materials Science , Nanjing Normal University , Nanjing , Jiangsu 210023 , P. R. China; c Davidson School of Chemical Engineering , Purdue University , West Lafayette , Indiana 47907 , USA

## Abstract

The internal free volume of porous materials diminishes upon mechanical compression, and such volume collapse can have chemical consequences. We report here the endothermic bond breakage in a metal-organic framework (MOF) during compression-induced collapse. Upon bulk compression at 1.9 GPa, the effective number for Zr–O bonds between Zr(iv) ions and carboxylate groups in UiO-66 decreased from 4.0 to 1.9, as determined by EXAFS, and the internal free volume was synchronously collapsed. Consistent with the EXAFS data, IR spectra confirmed conversion of *syn*–*syn* bridging carboxylates to monodentate ligation, thus establishing mechanochemical reactions induced by external compression of MOFs. Substantial mechanical energy (∼4 kJ g^–1^) was absorbed by UiO-66 nanocrystals during compression, as demonstrated from nanocompression of single crystals (600 nm) *in situ* during scanning electron microscopy, which establishes the potential application of MOFs as mechanical energy absorbers for hydrostatic and shock compression.

## Abstract 2

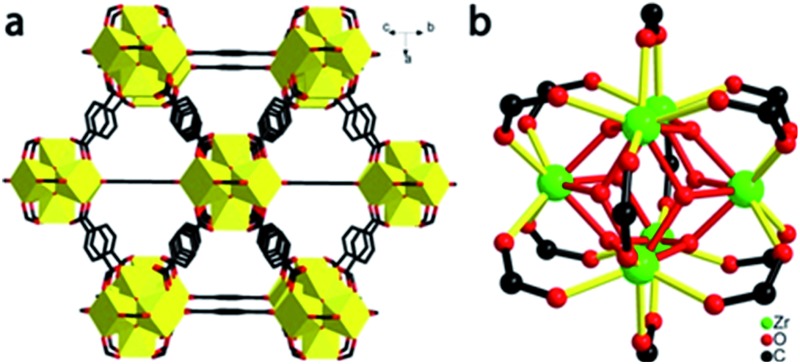
The internal free volume of porous materials diminishes upon mechanical compression, and such volume collapse can have chemical consequences.

## Introduction

The internal free volume of porous materials diminishes upon exposure to mechanical compression, with potential chemical consequences.^1^,^2^ Compared to traditional inorganic porous materials (*e.g.*, zeolites), metal organic frameworks (MOFs) can have much larger pore sizes and surface areas.^3^,^4^ The effects of pressure, compressional collapse, and the mechanochemistry^5^–^10^ of MOFs, however, have been little studied,^11^–^17^ whereas their promising applications in gas storage, separation and catalysis^18^–^20^ have been extensively explored over the past decade. When MOFs are subjected to strong compression, large negative Δ*V* and positive Δ*S* are expected due to the collapse of its internal free volume and the loss of crystallinity.^13^–^15^,^21^ Thus, one may speculate^14^,^22^,^23^ that MOFs might function as lightweight protective materials to absorb mechanical energy from shockwaves by their collapse and by endothermic bond breakage during their collapse.

**Fig. 1:**
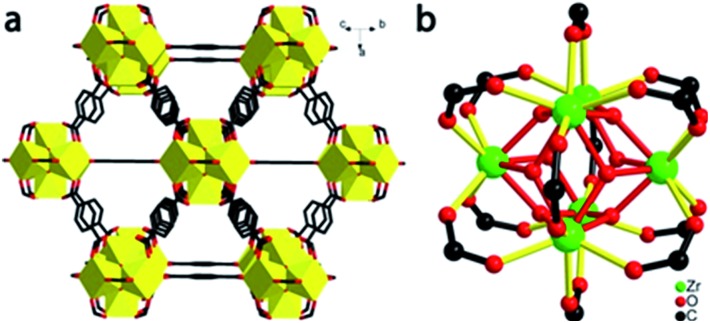
Structure of UiO-66. (a) Extended 3D structure, showing the bridging terephthalates ligated to zirconia clusters. (b) Coordination geometry around the Zr_6_O_4_(OH)_4_(O_2_CR)_12_ clusters.

In a prior study of a relatively compact framework solid (the Zn-imidazolate system called ZIF-8), we found that ZIF-8 lost porosity and long range order upon compression at ∼2 GPa, while its local structure around the bridging Zn ion (ZnN_4_) was maintained.^13^ In contrast to ZIF-8, we have now discovered with the more expansive structure of UiO-66,^24^ the first compression-induced endothermic bond breakage in MOF systems. Specifically, upon bulk compression of UiO-66 (Fig. 1) at 1.9 GPa, the effective number for Zr–O coordination bonds between Zr(iv) ions and carboxylate groups decreased by half and substantial mechanical energy was absorbed in the amorphization process.

## Materials and methods

### Synthesis of UiO-66 nanocrystals

UiO-66 nanocrystals were prepared using a previously reported method with some modifications.^25^ ZrCl_4_ (93 mg, 0.4 mmol) and terephthalic acid (67 mg, 0.04 mmol) were separately dissolved into 10 mL DMF. Acetic acid (2.8 mL, 50 mmol) was first added to the DMF solution of terephthalic acid with stirring, and the mixture was then poured into the DMF solution containing ZrCl_4_. The final mixture was transferred to a 100 mL Pyrex bottle and heated in oven at 120 °C for 24 h. The solid product was collected through centrifugation and washed twice with DMF and methanol, respectively. The product was immersed in methanol overnight to exchange remaining guest DMF solvates with methanol. After centrifugation, the product was heated to 200 °C under vacuum for 10 hours to fully remove all guest solvates. Desolvated UiO-66 was placed in a desiccator over CaSO_4_ until used; samples were shipped also over CaSO_4_ to prevent water sorption. TGA confirmed complete desolvation and the absence of sorbed methanol or water (ESI, Fig. S1†).

### Sample preparation and piston compression measurements

50 mg desolvated UiO-66 nanocrystals were placed in a 13 mm diameter die and vertically compressed by a hydraulic piston pelletizer (up to 25 ton load, *i.e.*, 1.9 GPa). After compression, UiO-66 crystals became pressed pellets. After releasing the pressure, the pellets were characterized by FT-IR, PXRD, XAS and BET analysis.

### Flat punch nanocompression experiment

For the *in situ* TEM nanocompression experiments, an individual desolvated UiO-66 nanocrystal was placed on the silicon wedge holder with a ∼1.0 μm flat face. The UiO-66 nanocrystal was positioned on the 111 facet (*i.e.*, diamond shaped projection). The punch (a flat square surface with a 2 μm edge length) compressed the crystal at 1 nm s^–1^ under displacement control mode. The morphological change was filmed by video TEM and correlated to the displacement of the piston. The applied force and the displacement of the piston were recorded by the transducer. Each data point in Fig. 6 represents a single experiment on a single nanocrystal; some of the scatter derives from the minor differences in size and contact surface among the nanocrystals; the smooth line is meant only as a guide to the eye.

### Instrumentation

Scanning electron microscopy (SEM) was performed using a Hitachi S-4800 field emission microscope at an accelerating voltage of 10 kV. The samples were prepared on Si wafers, then sputtered with a very thin layer of Au/Pd. BET surface area measurements were performed with a Nova 2200e, Quantachrome Instruments. FT-IR was performed on a Perkin-Elmer SpectrumBX instrument fitted a SensIR Technologies DuraSampleIR II ATR unit. Transmission electron microscopy (TEM) was performed on a 200 kV JEOL LaB6 TEM. The *in situ* video was captured using a Hysitron PI95 TEM Picoindenter accessory, Hysitron Corporation, Minneapolis, MN.

### XANES and EXAFS data collection and analysis

X-ray absorption measurements were acquired on the bending magnet beam line of the Materials Research Collaborative Access Team (MRCAT, sector 10) at the Advanced Photon Source, Argonne National Laboratory. Photon energies were selected using a water-cooled, double-crystal Si(111) monochromator, which was detuned by approximately 50% to reduce harmonic reflections. Measurements were made in transmission mode using 25 vol% argon with 75 vol% nitrogen in the incident ionization chamber (10% absorption at 18 300 eV) and a mixture of *ca.* 95 vol% argon with 5 vol% nitrogen in the transmission ionization chamber (30% absorption at 18 300 eV). Data points were acquired in six separate regions (energies relative to the elemental Zr K-edge 17 998 eV): a pre-edge region (–250 to –50 eV, step size = 10 eV, dwell time = 0.1 s), a second pre-edge region (–50 to –20 eV, step size = 2 eV, dwell time = 0.1 s), the XANES region (–20 to +30 eV, step size = 0.3 eV, dwell time = 0.3 s), an initial EXAFS region (+30 eV (2.80 Å^–1^) to 6 Å^–1^, step size = 0.07 Å^–1^, dwell time = 0.5 s), a second EXAFS region (6 Å^–1^ to 9 Å^–1^, step size = 0.07 k, dwell time = 1.0 s) and the final EXAFS region (9 Å^–1^ to 13 Å^–1^, step size = 0.07 k, dwell time = 1.5 s). The detector settling time was 0.1 s prior to each measurement with a beam size of 1.5 mm × 0.5 mm. Each sample (5.0 mg) was mixed with SiO_2_ (25 mg), and then the mixture was grinded well with mortar and pestle. 30 mg of the mixture was pressed into a cylindrical sample holder consisting of six wells with a radius of 2.0 mm, forming a self-supporting wafer. The sample holder was placed in a quartz tube (1-in. OD, 10-in. length) sealed with Kapton windows by two Ultra-Torr fittings and then used for transmission mode measurement. The edge energy of the X-ray absorption near edge structure (XANES) spectrum was determined from the inflection point in the edge, *i.e.*, the maximum in the first derivative of the XANES spectrum. Background removal and normalization procedures were carried out using the Demeter software package 0.9.24 using standard methods of Athena. Standard procedures based on Artemis software were used to extract the extended X-ray absorption fine structure (EXAFS) data. The crystal structure (CCDC 733458) of UiO-66 was used as the fitting model, and the amplitude reduction factor was obtained by fixing the coordination number of the uncompressed UiO-66. The same amplitude reduction factor was then used to fit the compressed samples. The coordination parameters were obtained by a least square fit in *R*-space of the nearest neighbor, *k*^2^-weighted Fourier transform data. The data fit equally well with either *k*^1^ or *k*^3^ weightings.

## Results and discussion

### Crystal structure and preparation of UiO-66

The structure of UiO-66 (Fig. 1) has octahedral Zr_6_O_4_(OH)_4_ clusters that are *syn*–*syn* bridged by 12 carboxylate groups from bridging terephthalates.^24^ The Zr_6_-octahedra are capped alternatively by μ_3_-O or μ_3_-OH groups, which connect each Zr(iv) to four other Zr(iv), with a Zr···Zr distance of 3.513(1) Å. There are two types of Zr–O bonds in UiO-66: the Zr–carboxylate (Zr–O_COO_, 2.232(2) Å) and the Zr-bridging O/OH (Zr–O_μ_3_-O_, 2.108(3) Å).

**Fig. 2:**
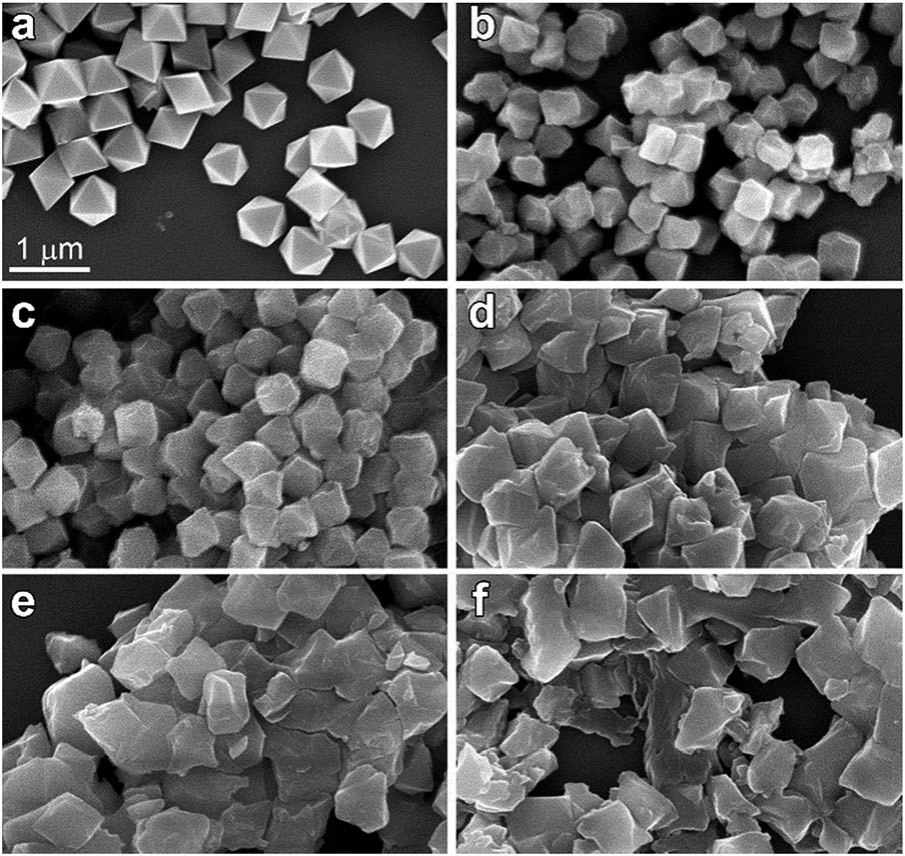
SEM images of UiO-66 nanocrystals (a) as prepared, and after applied compressive pressures of (b) 0.4 GPa, (c) 0.8 GPa, (d) 1.1 GPa, (e) 1.5 GPa, and (f) 1.9 GPa.

UiO-66 nanocrystals were synthesized by a solvothermal method and desolvated, as per prior literature with minor modification.^25^ The UiO-66 nanocrystals have a well-defined octahedral morphology with an average size of 600 nm (ESI, Fig. S2†). Desolvated UiO-66 (50 mg) was placed in a 13 mm diameter stainless steel die and axially compressed by a hydraulic piston pelletizer at applied pressures up to 1.9 GPa; after release of the applied load, irreversible changes in morphology and substantial amorphization were observed (Fig. 2 and S3†). The UiO-66 nanocrystals underwent deformation and agglomeration with increasing applied pressure, converting from an initially well-defined octahedral morphology to irregular flattened agglomerates (Fig. 2).

### Characterization of UiO-66 after compression and release

The BET surface area of the UiO-66 dramatically decreased upon compression, from 1050 m^2^ g^–1^ for the initial nanocrystal to 76 m^2^ g^–1^ after compression at 1.9 GPa (Table 1), similar to our observations with ZIF-8.^13^ The powder X-ray diffraction (PXRD) pattern of the sample after compression under applied pressures ≤0.4 GPa was consistent with uncompressed UiO-66 (Fig. S3†); after compression above 0.4 GPa, however, the PXRD patterns were weak, consistent with substantial and irreversible amorphization upon pore collapse.

**Table 1:** BET Surface areas and pore volumes for UiO-66 and ZIF-8 crystals as a function of applied compression pressure

Sample	BET Surface area (m^2^ g^–1^)		Pore volume (cm^3^ g^–1^)	
	ZIF-8	UiO-66	ZIF-8	UiO-66
As prepared	1340	1050	0.66	0.54
After 0.4 GPa	1110	800	0.54	0.38
After 0.8 GPa	850	490	0.40	0.23
After 1.1 GPa	510	210	0.24	0.09
After 1.5 GPa	440	130	0.19	0.05
After 1.9 GPa	250	76	0.10	0.03

**Fig. 3:**
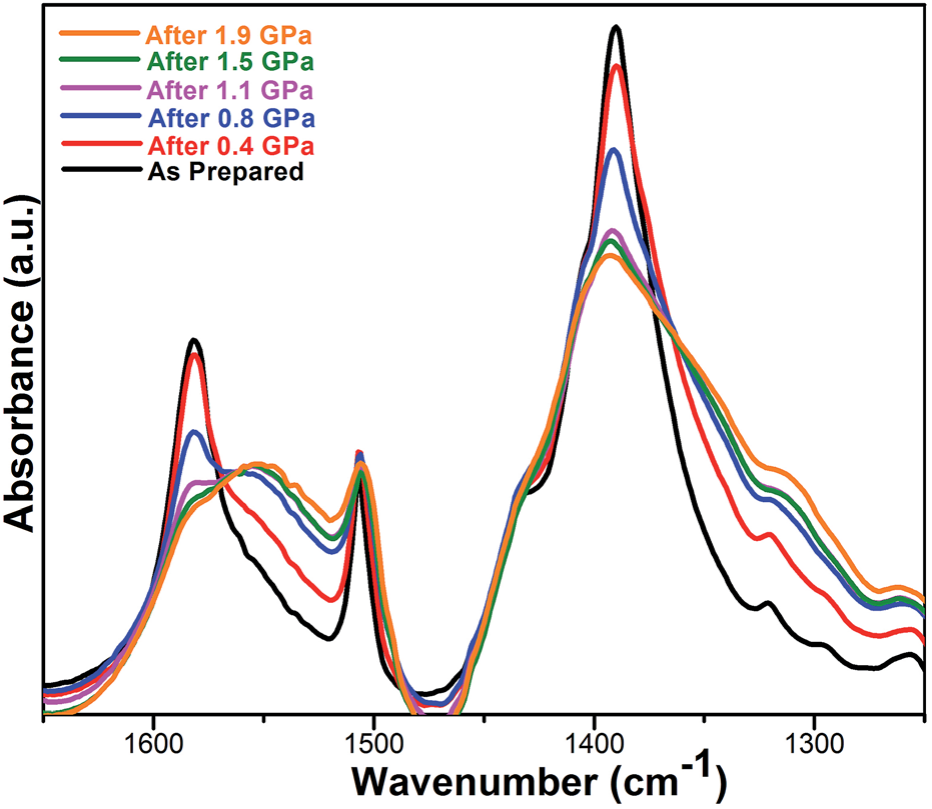
FT-IR spectra of UiO-66 over the range of 1650–1250 cm^–1^ after compression and release. All absorbances were normalized to the peak at 1019 cm^–1^ (*cf.* ESI Fig. S4†).

**Scheme 1:**
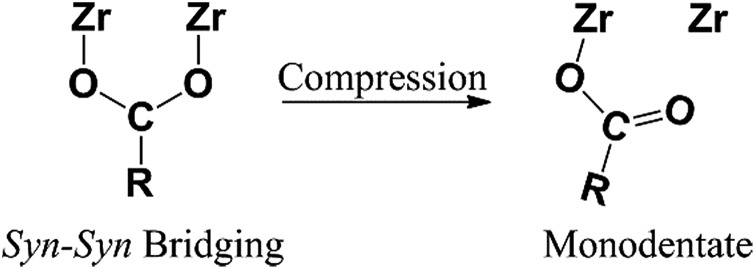
Change in coordination mode of the bridging carboxylate within each Zr–O cluster in the UiO-66 upon compression.

To understand better the effects of compression on the solid state structure, we examined the FT-IR spectra of the UiO-66 samples as a function of applied pressure. The spectrum of uncompressed UiO-66 (Fig. 3 and S4†) is consistent with previous reports:^24^ the strong sharp bands at 1578 cm^–1^ and 1390 cm^–1^ are assigned to the asymmetric and symmetric stretch of the carboxylate groups from the terephthalate, respectively, consistent with the *syn*–*syn* bridging mode of the carboxylate group in other complexes (Scheme 1).^26^

After compression at 0.4 GPa, most absorption bands in the FT-IR spectrum remained unchanged (Fig. S4†) except for bands from the carboxylate group (Fig. 3). The carboxylate stretch at 1578 cm^–1^ broadened and a shoulder band at 1550 cm^–1^ appeared, which is a characteristic asymmetric stretch for monodentate coordinated carboxylates (Scheme 1).^26^ This change indicates that the coordination mode of carboxylate group in UiO-66 has partially transitioned from the *syn*–*syn* bridging to a monodentate coordination mode, and the bond between the carboxylate and Zr(iv) (Zr–O_COO_) has been substantially broken. Combining these results with PXRD after compression at 0.4 GPa, the long range order of UiO-66 has been maintained, but the local coordination environment around Zr(iv) has been partially changed.

As the pressure of the compression was increased, the shoulder band at 1550 cm^–1^ became enhanced and the band at 1390 cm^–1^ greatly broadened. The other major bands remained the same although slight broadening was observed (Fig. S4†). After compression at 1.9 GPa, the shoulder band intensity at 1550 cm^–1^ becomes comparable to the stretching band at 1578 cm^–1^, indicating more Zr–O_COO_ bonds between carboxylate and Zr(iv) have been broken with increasing applied pressure.

### X-ray absorption spectra of the UiO-66 after compression and release

**Fig. 4:**
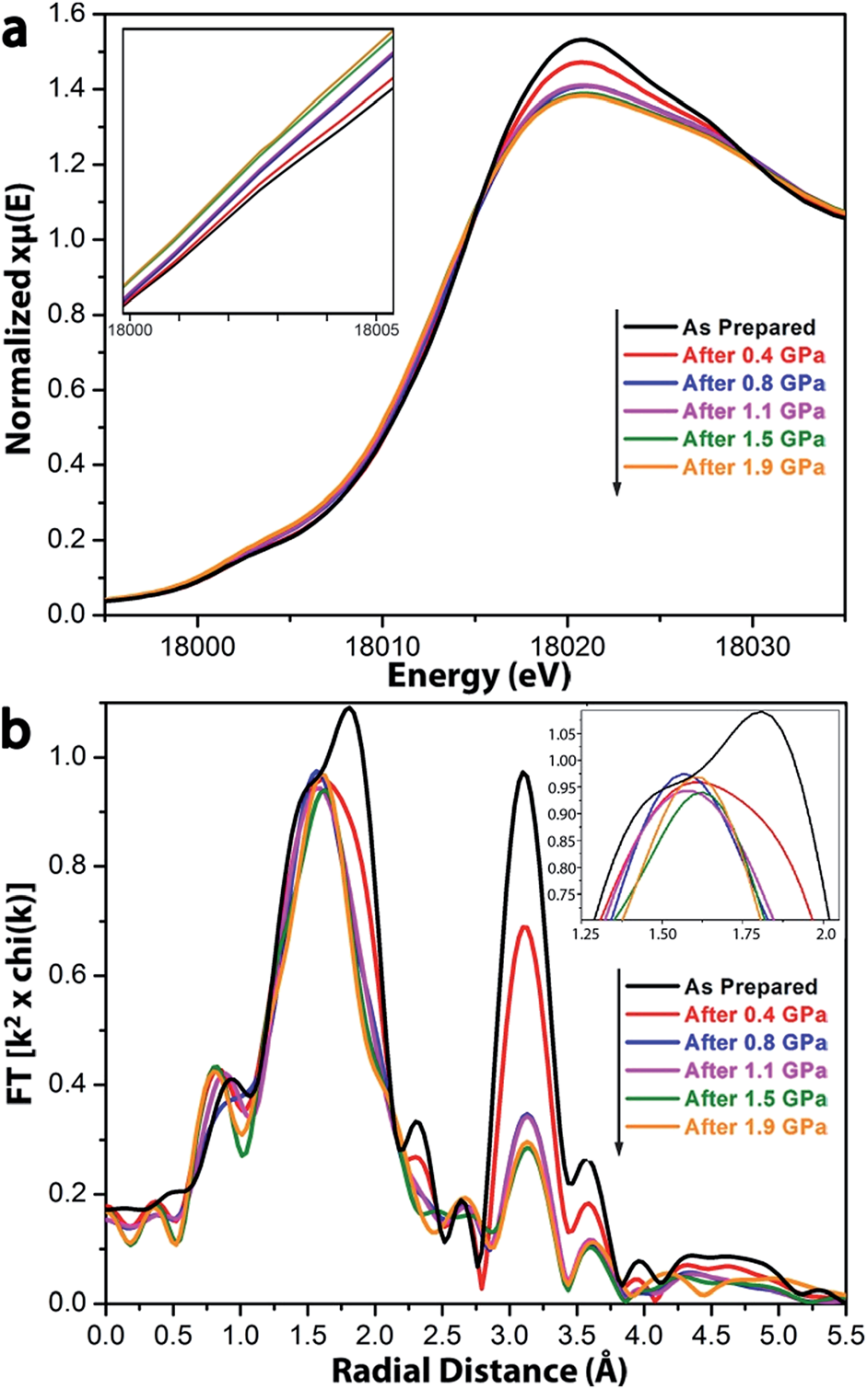
(a) XANES spectra for UiO-66 samples as a function of compression at applied pressures ranging from 0 to 1.9 GPa. Inset shows the zoomed in pre-edge region. (b) Magnitude of the *k*^2^-weighted Fourier transform of the EXAFS spectra of UiO-66 as prepared and after compression at various applied pressures followed by release. Insets are magnifications of the region shown.

To understand quantitatively the bond breakage in UiO-66 after compression, X-ray absorption spectroscopy (XAS) was employed to probe the local coordination environment around Zr(iv) ions (Fig. 4).^27^ The oxidation state and coordination geometry were obtained from the X-ray absorption near edge structure (XANES), and information regarding the coordination numbers can be extracted from the extended X-ray absorption fine structure (EXAFS).^28^,^29^

As suggested by the XANES spectra (Fig. 4a), the oxidation state of Zr remains +IV after compression at 0.4 through 1.9 GPa. The systematic increase of the feature at 18 003 eV and decrease at 18 020 eV as a function of extent of compression indicate a decrease in the local symmetry around Zr(iv) ions.^27^ The feature at 18 003 eV may be assigned as the dipole-forbidden 1s–4d transition. In the uncompressed UIO-66, the Zr ions are in a relatively symmetric Zr_6_-octahedra, which has a little d–p mixing, so the 1s–4d transition shows a very small peak. After compression, the geometry around Zr is not preserved, and greater local asymmetry gives more 4d–5p mixing and leads to an increase in intensity of the transition. The feature at 18 020 eV may be assigned to the dipole-allowed 1s–5p transition; the observed decrease of this feature after compression is also consistent with increased 4d–5p mixing.

In the EXAFS spectrum for the uncompressed UiO-66 (Fig. 4b), two major peaks were observed. The first corresponds to the nearest neighbors of the Zr(iv) ion, *i.e.*, the Zr–O shell; overlapping features at 1.5 Å and 1.8 Å (phase uncorrected distances) correspond to the Zr–O_μ_3_-O_ and Zr–O_COO_ bonds, respectively, which have different bond lengths (Zr–O_μ_3_-O_ < Zr–O_COO_). The second intense peak at 3.1 Å is ascribed to the next–nearest neighbors, *i.e.*, the Zr···Zr shell.

After compression, EXAFS spectra show changes in the coordination environment around Zr(iv) in UiO-66 (Fig. 4). The peaks at 1.8 Å and 3.1 Å dramatically decrease with increasing compression, which indicates the loss of Zr–O_COO_ bonds and Zr···Zr contacts, consistent with the observations from the FT-IR spectra (Fig. 3). In contrast, the peak intensity at 1.5 Å (from the Zr–O_μ_3_-O_ bonds) stays the same regardless of the extent of compression, which implies that the inner Zr–O_μ_3_-O_ bonds were not affected by compression.

**Fig. 5:**
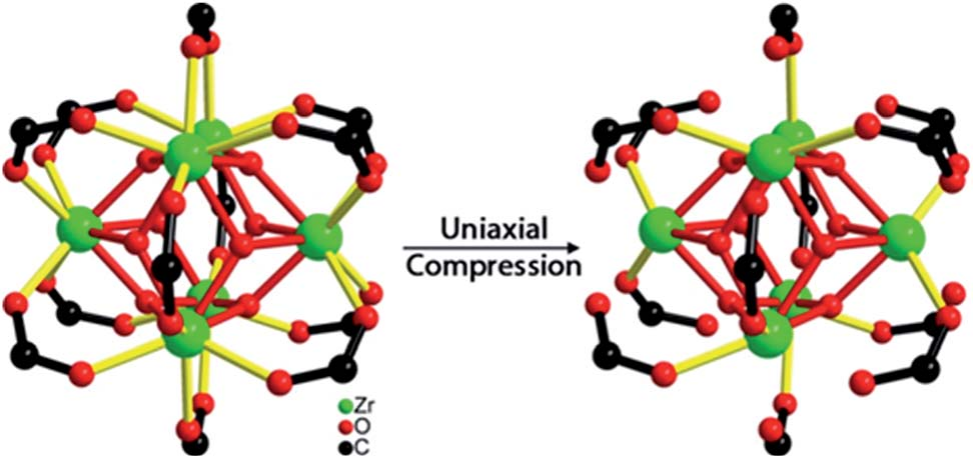
After compression, the effective number of Zr–carboxylate oxygen (Zr–O_COO_) bonds (shown in yellow) for each Zr(iv) ion decreased from 4 to ≈2.

The extent of bond breakage of Zr–O coordination that occurs during compression has been measured from our EXAFS results. Fitting the *R*-space EXAFS spectra permitted a quantitative modeling of the bond breakage that occurs during pressurization (Fig. S5–S8†), and the results are summarized in Table 2. For UiO-66 before compression, the fitting results are fully consistent with the XRD single-crystal structure: each Zr(iv) has 4 Zr–O_COO_ bonds, 4 Zr–O_μ_3_-O_ bonds, and 4 Zr···Zr next nearest neighbor contacts. For UiO-66 after pressure treatment at 0.4 GPa, the effective number of Zr–O_COO_ bonds and Zr···Zr scatterers decreased to 3.0 and 2.5 per Zr(iv) ion, respectively. As compression is increased to 1.9 GPa, the effective number of Zr–O_COO_ bonds and Zr···Zr scatterers monotonically decreases to 1.9 and 2.1 per Zr(iv) ion, respectively (Fig. 5). This change coincides with the loss in pore volume (Table 1). The EXAFS spectra indicate that upon pressure-induced amorphization and pore collapse, roughly half of the initial Zr–O_COO_ bonds appear to have been broken: the total coordination number of the Zr(iv) ions goes from 8 to roughly 6 (*i.e.*, 4 unchanged Zr–O_μ_3_-O_ bonds with the number of Zr–O_COO_ bond decreased from 4 to 1.9, as seen in Table 2).

**Table 2:** Summary of EXAFS fitting results^*a*^

Pressure (GPa)	Bond types	Coordination numbers^*b*^	Bond length^*b*^ (Å)
UiO-66, as-prepared	Zr–O_COO_	4.0	2.27
	Zr–O_μ_3_-O_	4.0	2.12
	Zr···Zr	4.0	3.53
0.4	Zr–O_COO_	3.0	2.29
	Zr–O_μ_3_-O_	4.0	2.14
	Zr···Zr	2.5	3.54
0.8/1.1^*c*^	Zr–O_COO_	2.2	2.30
	Zr–O_μ_3_-O_	4.0	2.14
	Zr···Zr	2.4	3.54
1.5/1.9^*c*^	Zr–O_COO_	1.9	2.33
	Zr–O_μ_3_-O_	4.0	2.16
	Zr···Zr	2.1	3.54

^*a*^FT range: 3.5–12.5 Å^–1^; fitting range: 1.25–3.48 Å.

^*b*^Estimated errors in coordination numbers are approximately ±5% relative and ±10% absolute; errors in bond lengths are ±0.02 Å.

^*c*^In both XANES and EXAFS spectra, UiO-66 samples after compression at 0.8 and 1.1 GPa are very similar, as are the spectra after compression at 1.5 and 1.9 GPa.

The FT-IR data are consistent with the EXAFS observations of bond breakage. The observation of well-defined isosbestic points in the FT-IR spectra after compression (Fig. 3 and S4†) suggests the presence of only two species: the initial eight-coordinate Zr ions with bridging carboxylates and the putative six-coordinate monodentate carboxylate–Zr species formed during amorphization.

The bond length measured from the EXAFS spectra were generally consistent with similar structures determined by single-crystal XRD. The fitted bond lengths of Zr–O_COO_, Zr–O_μ_3_-O_ and Zr···Zr were 2.27 Å, 2.12 Å and 3.53 Å, respectively, in agreement with those from the XRD single-crystal structure^24^ of UiO-66 (Table 2). The average fitting bond length of the existed Zr–O_COO_ bond increases as the applied pressure is increased and as the transition from *syn*–*syn* bridging to monodentate ligation occurs. After application of 1.9 GPa during compression, the average fitting bond length of Zr–O_COO_ bond increased slightly from 2.27 Å to 2.33 Å.

Prior literature on structural consequences of pressure-induced amorphization of MOFs is limited. Theoretical modeling of MOFs have very recently predicted substantial structural rearrangement after compression,^23^,^30^ but this is the first time that compression-induced bond breakage in MOFs has been directly observed. In ball-milling of crystalline UiO-66 sample, there is a similar phenomenon reported.^31^ The Zr_6_O_4_(OH)_4_ clusters appear to remain intact, but ∼6.8% of the total Zr–O_COO_ bond were broken during ball-milling, as attributed to a new feature in the solid state ^13^C NMR of the amorphized UiO-66. Given the difficulties in estimating effective pressures during ball-milling and the random orientation of collision of crystals with the balls, a more detailed comparison is not possible.

### Flat punch nanocompression experiment

**Fig. 6:**
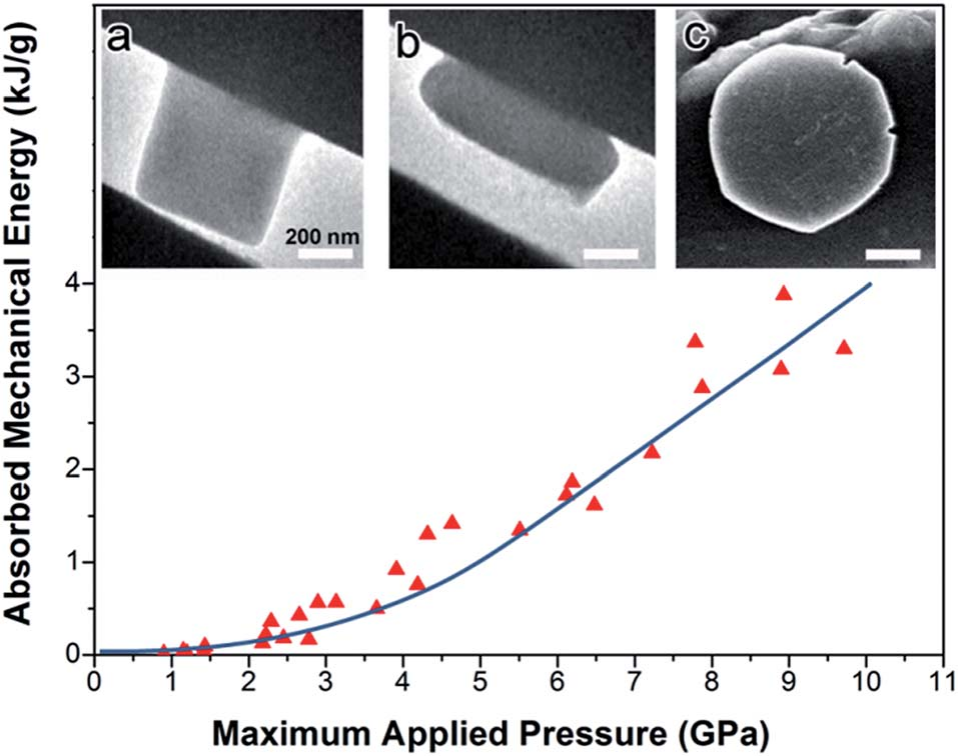
Absorbed mechanical energy (*i.e.*, inelastic) as a function of maximum applied pressure for individual UiO-66 nanocrystals in the flat piston compression experiments. Insets: (a) side view TEM of a single crystal of UIO-66 mounted on the silicon wedge holder before compression; (b) same crystal after compression to 2000 μN, and (c) top view, SEM of a compressed nanocrystal resembling a flattened pancake.

To explore the energetics of the compression of UiO-66 nanocrystals, flat punch nanocompression experiments were carried out inside an electron microscope (Fig. 6; ESI Fig. S9–S11†).^13^,^15^ Fig. 6 presents the absorbed mechanical energy by UiO-66 single nanocrystals under uniaxial compression. The details of the calculation of absorbed mechanical energy are presented in ESI Fig. S9 and S10.† Briefly, the *in situ* TEM images during the nanocompression experiments are used to calculate the mass of the individual nanocrystals, their area of contact against the compressing punch and anvil, and (combined with the measured applied force from the nanocompressor device) the effective compression pressure. The absorbed mechanical energy per gram is derived from integration of the load–displacement curves during the loading and unloading process.

At lower applied pressure (Fig. 6, <2 GPa), only a modest amount of mechanical energy (∼0.1 kJ g^–1^) was absorbed by individual UiO-66 nanocrystals during essentially elastic compression (which is comparable to the energy consumed by the reversible elastic deformation of another MOF, MIL-53 (ref. 32)).

As the pressure applied to UiO-66 nanocrystals was increased (to as high as ∼10 GPa), the total absorbed mechanical energy increases substantially (Fig. 6). The energy absorbed by UiO-66 is as large as ∼4 kJ g^–1^. For calibration and context, the energy density of explosives is several kJ g^–1^: *e.g.*, TNT (2,4,6-trinitrotoluene) generates ∼4.2 kJ g^–1^ during explosion.^33^ Thus, on a gram for gram basis, the endothermic chemical reactions associated with the collapse of this MOF are comparable in magnitude to the exothermicity of a typical explosive. In both cases, the release or absorption of energy is essentially irreversible. We do not mean to imply at this time that MOFs will be capable of energy absorption from shock waves; such a conclusion will have to await ongoing studies of the effects of shock impact on MOFs.

The apparent applied pressure used during piston compression of bulk UiO-66 samples may not be directly comparable to the applied pressure used in the well-defined uniaxial compression of single nanocrystals because of possible effects of nanocrystal–nanocrystal interactions (*e.g.*, shear stress, non-uniform contacts, non-uniaxial effects, *etc.*) during compression of the bulk sample. These interparticle interactions make the effects of the apparent applied pressure during bulk compression much greater than the equivalent applied pressure in the single crystal nanocompression experiments.

### Absorption of mechanical energy

Compression-induced bond breakage provides a potential application of MOFs as mechanical energy absorbers for hydrostatic and shock compression. In the complex of Zr_4_O_2_(CHF_2_COO)_12_, the bridging carboxylate groups are in the same *syn*–*syn* coordination mode to Zr(iv) ions as in UiO-66, and the Zr–O_COO_ bond between the carboxylate group and Zr(iv) ions has a bond length of 2.23 Å,^34^ which is very close to the Zr–O_COO_ bonds in UiO-66. According to a DFT calculation,^35^ the binding energies for CHF_2_COO^–^ to the Zr_4_-cluster is –143 kcal mol^–1^, *i.e.*, the bond enthalpy for each Zr–O_COO_ bond is approximately half, *i.e.*, 72 kcal mol^–1^, which should be comparable to the Zr–O_COO_ bond energy in UiO-66.

As discussed earlier, after compression of bulk UiO-66 to 1.9 GPa, the EXAFS data for UiO-66 show that two Zr–O_COO_ bond per Zr(iv) ion have been broken. Each UiO-66 unit cell has six Zr(iv) ions (Zr_6_O_4_(OH)_4_(BDC)_12_ where BDC = terephthalate and a unit cell molecular weight of 1662 g mol^–1^). Let us assume a simple model in which all six Zr(iv) ions, which were initially each eight-coordinate (four Zr–O_COO_ and Zr–O_μ_3_-O_ bonds), are converted to six-coordinate Zr(iv) (two Zr–O_COO_ and four Zr–O_μ_3_-O_ bonds). Bond breakage is endothermic, and in this case, an estimate of the absorbed energies per gram from the endothermic bond breakage of UiO-66 after compression at 1.9 GPa can be approximated as
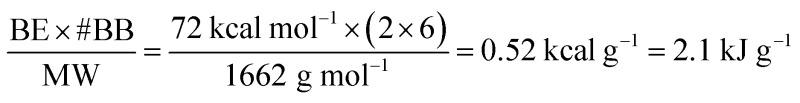
where BE is the bond enthalpy of the bonds being broken and #BB is the number of broken bonds per unit cell, and MW is the molecular mass of the unit cell. This simple model, then, accounts for a significant fraction of the total absorbed mechanical energy during the compression of UiO-66 (Fig. 6).

Thus, if two Zr–O_COO_ coordination bonds per Zr ion are broken (consistent with the EXAFS data), each gram of UiO-66 after the pressurization to 1.9 GPa would have converted ∼2.1 kJ mechanical energy to chemical energy (assuming no compensating increase in other bonding in the amorphized structure). This predicted conversion of mechanical energy (∼2.1 kJ g^–1^) is about 70 times the experimentally observed energy absorbed by reversible elastic deformation of MOF MIL-53 (maximum ∼0.033 kJ g^–1^), and more than 200 times the energy absorbed during intrusion experiments on mesoporous silica (∼4 to 12 × 10^–3^ kJ g^–1^).^32^,^36^,^37^ One may conclude that Zr–O_COO_ bond breakage is the primary component of the large energy absorbed during compressional collapse of UiO-66.

### Comparison to other MOFs

Not all MOFs will necessarily break bonds during compression and void collapse. In a prior study, we found that another framework solid, ZIF-8 (zeolitic imidazolate framework-8), also lost porosity and long range order upon static compression up to ∼2 GPa; in contrast, however, ZIF-8 maintained its local structure around the bridging Zn ion (ZnN_4_).^13^ We suggest that this is probably due to the relative compactness of the 2-methylimidazole ligand and the relatively dense structure of ZIF-8 compared to UiO frameworks. Indeed, our choice of UiO-66 for this study was predicated on its longer bridging ligand (terephthalate) and larger pore size, in addition to the extremely high chemical and thermal stability of the UiO frameworks.^38^,^39^ Moreover, unlike the free rotation between the 2-methylimidazole to Zn(ii) ions under compression, the orientation between the ligand terephthalate and Zr_6_O_4_(OH)_4_ clusters was fixed by the bidentate *syn*–*syn* bridging of the carboxylates, again leading one to expect greater rigidity in UiO-66 compared to ZIF-8.^31^,^39^,^40^

Our preliminary study on the mechanical property of isoreticular UiO MOFs under the compression has shown that the plasticity and endothermicity during the deformation of UiO MOFs displayed a surprising potential for absorption and dissipation of mechanical shock.^14^ The absorbed mechanical energy was calculated from individual UiO nanocrystals during one cycle of loading and unloading, as also used in this study, for a range of different bridging dicarboxylate bridges. The work here for the high pressure treatment of bulk UiO-66 samples further confirmed the capacity of the absorption and dissipation of mechanical energy.

The mechanical property of UiO MOFs also depends on the defects in the structure.^41^ Defects in UiO-66 solids are due primarily to vacancies in the coordination of the bridging terephthalates to the Zr_6_O_4_(OH)_4_ clusters when water molecules or added “modulators” (as HCOOH or CH_3_COOH, which aid in crystallization) replace terephthalate during crystal growth.^42^,^43^ Adsorption and thermal–mechanical property can be tuned by the density of defects in the structure.^41^,^44^ As discussed elsewhere,^15^ we were able to quantify such defects in UiO-66 by digesting desolvated UiO-66 in H_2_SO_4_/DMSO-*d*_6_ solution and analyzing the relative amount of dicarboxylate ligand and monocarboxylate modulators by ^1^H-NMR (the measured contamination^15^ of acetic acid to terephthalic acid in these UiO-66 samples is 5%). Indeed, the mechanical stiffness of UiO-66 depends very strongly on the defect concentration in the solid.^15^

The Zr–O_COO_ bonds in UiO-66 break as the collapse of the internal porosity occurs at relative low applied pressure (0.4 GPa). This mechanical behavior is rather different than that of another porous MOF, MIL-53.^32^ The structure of MIL-53 is built from trans corner sharing octahedra MO_4_(H_2_O)_2_ (M = Al(iii) or Cr(iii)) chains linked to each other by terephthalates.^45^,^46^ Upon compression up to 1.0 GPa, MIL-53 underwent a reversible structure transition, from a large pore phase to narrow pore phase, where the coordination bonds were maintained.^32^ It may be that MIL-53 at higher compressive pressures would also undergo irreversible bond breakage.

There are three responses that porous materials can have to compression from applied pressure: (1) in flexible or soft porous materials, the internal free volume collapses and densification occurs, generally with local structure maintained;^22^,^32^,^36^ for example, ZIF-8 (ref. 13) or MIL-53;^32^ in hard materials, (2) more substantial structural change may be associated with pressure-induced phase transitions;^47^ and as we have seen here, in rigid porous materials, (3) bond breakage at rigid sites in the structure may occur. The orientation of the terephthalate ligands in UiO-66 is fixed by the Zr_6_-linker, and therefore the Zr–O bonds between the carboxylate groups and Zr(iv) are vulnerable to breaking. In all three mechanisms, porous materials store the mechanical energy into the framework during compression, but endothermic bond breakage is likely to be a far larger energy contribution. The first two responses may or may not be reversible upon pressure release, but the third may be generally expected to be irreversible.

## Conclusions

In this work, we have explored the subtle relationship between the mechanical properties and structures of metal-organic framework solids and discovered mechanochemical^5^–^8^ reactions in MOFs during pressure-induced amorphization. We find compression-induced bond breaking in a MOF as demonstrated through EXAFS and confirmed by IR spectra. The bond breakage is a consequence of changes forced upon the extended structure of MOFs as pore collapse occurs. Collapse of the pores in UiO-66 forced the breakage of Zr–O bonds between the bridging terephthalates to the Zr_6_O_4_(OH)_4_ clusters. We have quantitatively investigated the nature of bond breakage as a function of the compressional pressure and provided structural information about the transition during compression. The mechanochemistry of MOFs is strongly endothermic and by direct observation of morphological changes of single crystals of UiO-66 by SEM during nanocompression experiments, we found that substantial energy was irreversibly absorbed in the solids during collapse, comparable in magnitude to the energy released by typical explosives.

## Conflicts of interest

The authors declare no competing financial interests.

## Supplementary Material

Supplementary information
