# Supplementary material for: Bond breakage under pressure in a metal organic framework
Source: Chem Sci. 2017 Oct 9;8(12):8004–11. doi: 10.1039/c7sc03786d (PMC5853553; doi:10.1039/c7sc03786d)
Supplement: Supplementary file 1 [file SC-008-C7SC03786D-s001.pdf]

Electronic Supplementary Material (ESI) for Chemical Science.
This journal is © The Royal Society of Chemistry 2017

Electronic Supporting Information

Bond Breakage under Pressure in a Metal Organic Framework

Zhi Su[ab], Yu-Run Miao[a], Guang-Hui Zhang[c], Jeffrey T Miller[c] and Kenneth S. Suslick*[a]

[a]Department of Chemistry, University of Illinois at Urbana-Champaign, Urbana, Illinois 61801, USA
[b]School of Chemistry and Materials Science, Nanjing Normal University, Nanjing, Jiangsu, 210023, P.R. China
[c]Davidson School of Chemical Engineering, Purdue University, West Lafayette, Indiana, 47907 USA

Email: Kenneth S. Suslick, ksuslick@illinois.edu

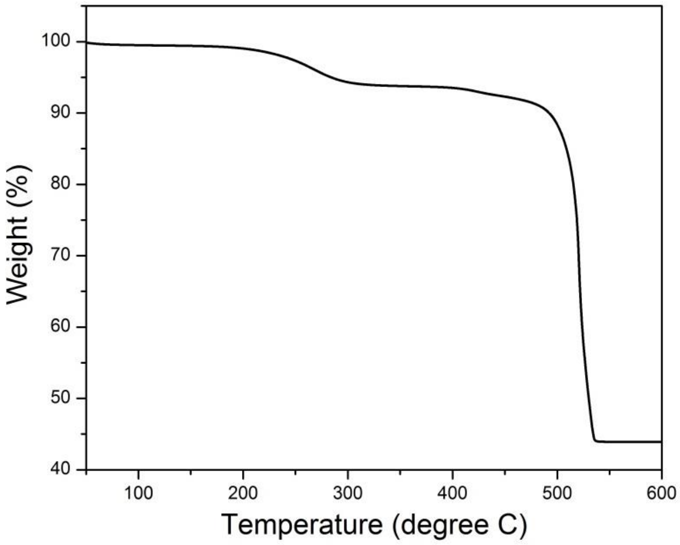

Figure S1. TGA of UiO-66 after desolvation from 50-600 °C. The absence of weight loss below 200°C demonstrates that there is no significant amount of adsorbed water or methanol in this material. For comparison to UiO-66 with water adsorbed, cf. ref 1.

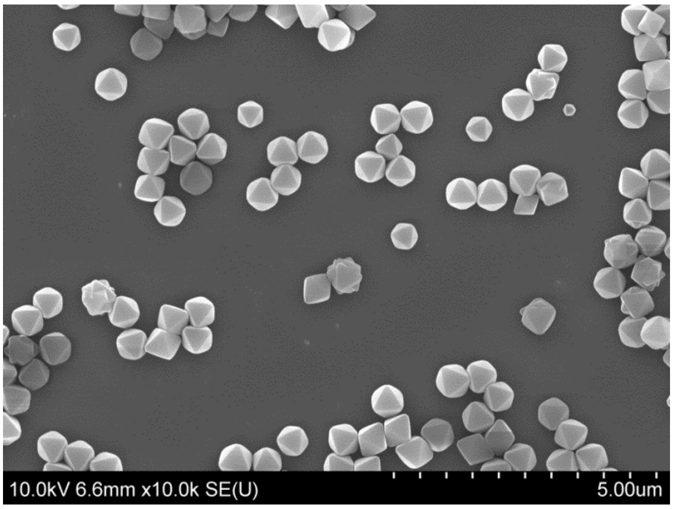

Figure S2. SEM image of the UiO-66 nanocrystals as synthesized.

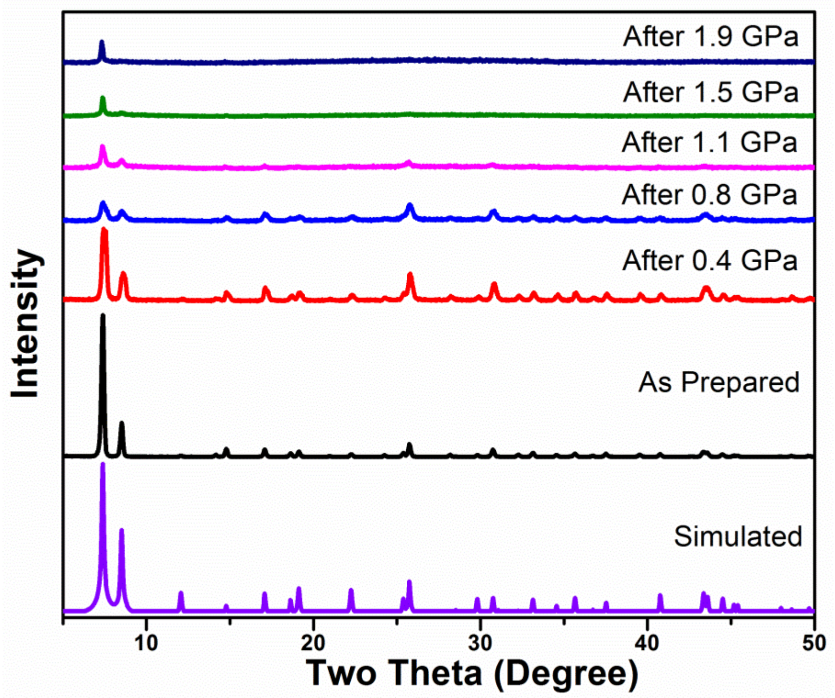

Figure S3. PXRD of UiO-66 nanocrystals after compression by a hydraulic piston followed by pressure release. Identical conditions were used for obtaining all PXRD patterns so the intensities of the diffraction peaks can be quantitatively compared. The SEM images (Figure 2) show that the morphology of UiO-66 nanocrystals endured deformation upon applied pressure, transitioning with increasing applied pressures from well-defined octahedra (<0.4 GPa) to irregular blocks (~1 GPa) to agglomerates (1.9 GPa).

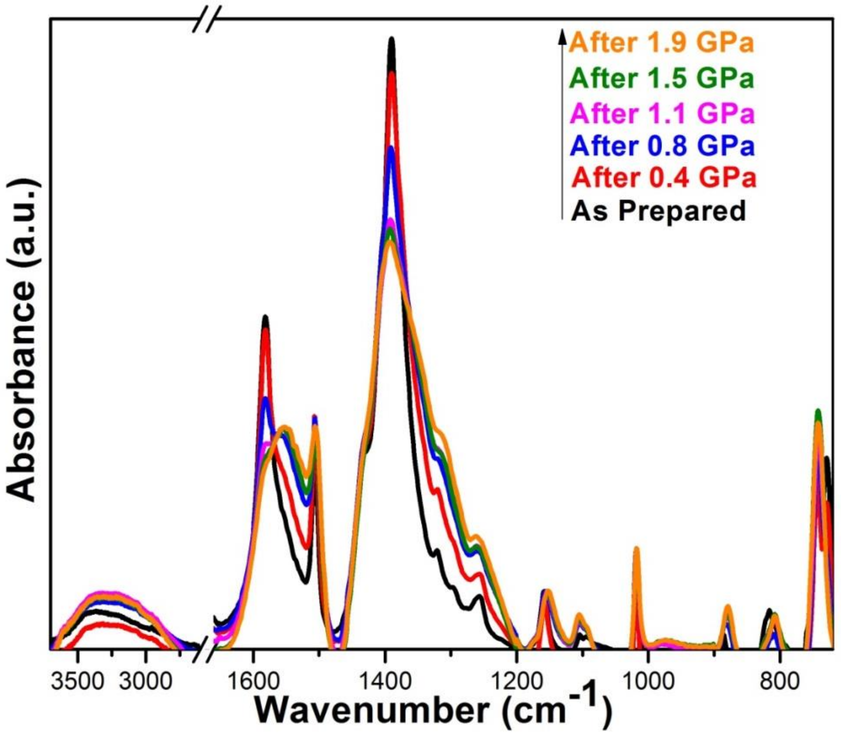

Figure S4. FT-IR spectra over the range of 3750 – 720 cm^{-1} of UiO-66 nanocrystals after compression and pressure release, and the absorbances have been normalized to the peak at 1019 cm^{-1}. Note the presence of well-defined isosbestic points, which is consistent with two species, the eight-coordinate bridging carboxylates and the putative six-coordinate monodentate carboxylate species discussed in the text.

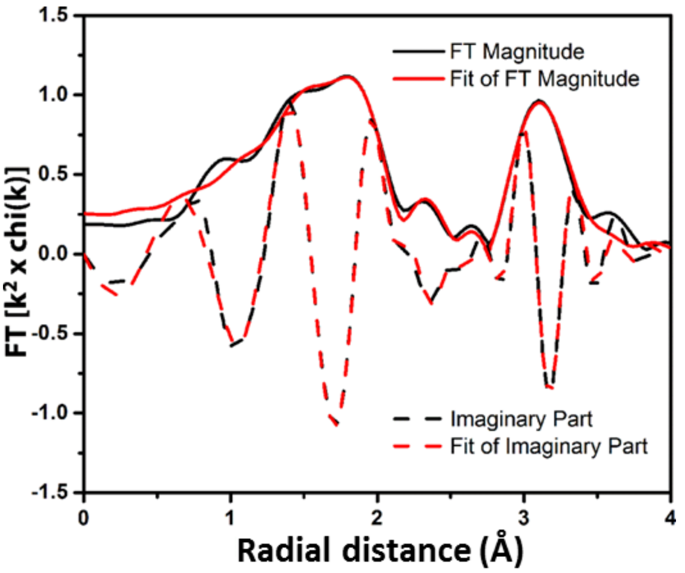

Scatterer	Zr-O_{coo}	Zr-$O_{\mu3\text{-}O}$	Zr···Zr
Coordination Number	4.0	4.0	4.0
Bond Distance (Å)	2.27	2.12	3.53
σ^2	0.002	0.005	0.004

Figure S5. Fitting results of the R-space EXAFS spectrum of UiO-66 as prepared (FT range: 3.5-12.5 $Å^{-1}$; fitting range: 1.25 -3.48 Å).

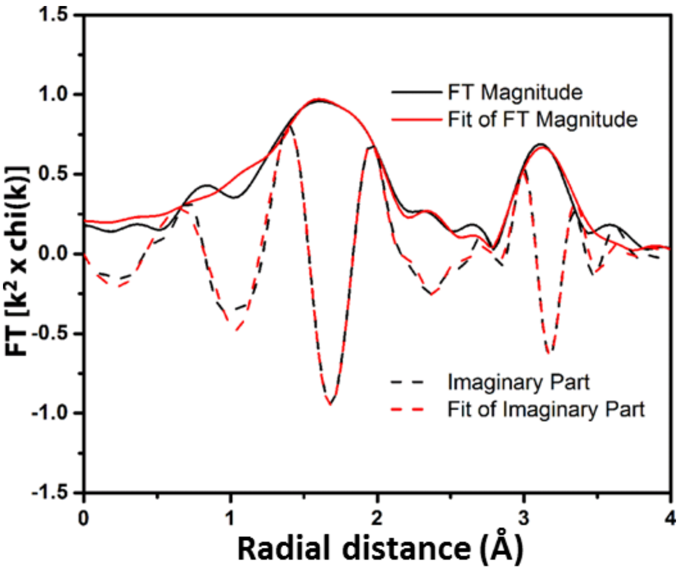

Scatterer	Zr-O_{coo}	Zr-$O_{\mu3\text{-}O}$	Zr···Zr
Coordination Number	3.0	4.0	2.5
Bond Distance (Å)	2.29	2.14	3.54
σ^2	0.003	0.006	0.004

Figure S6. Fitting results of the R-space EXAFS spectrum of UiO-66 after compression at 0.4 GPa (FT range: 3.5-12.5 $Å^{-1}$; fitting range: 1.25 -3.48 Å).

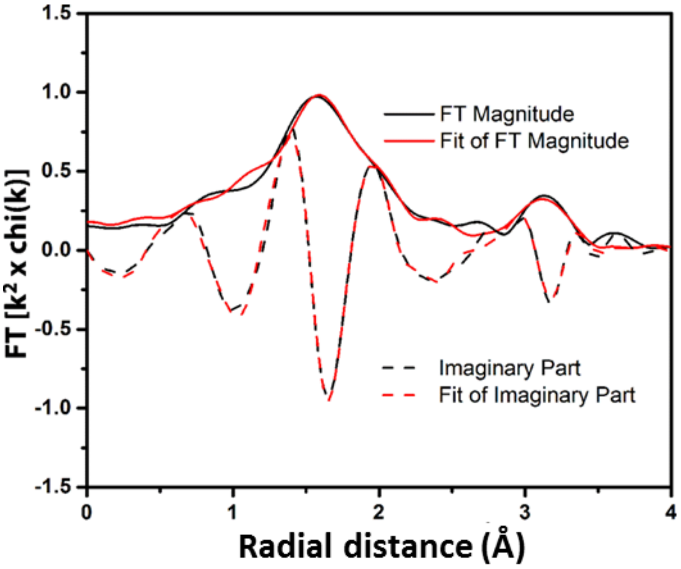

Scatterer	Zr-O_{coo}	Zr-$O_{\mu3\text{-}O}$	Zr···Zr
Coordination Number	2.2	4.0	2.4
Bond Distance (Å)	2.30	2.14	3.54
σ^2	0.002	0.004	0.010

Figure S7. Fitting results of the R-space EXAFS spectrum of UiO-66 after compression at 0.8 GPa (FT range: 3.5-12.5 $Å^{-1}$; fitting range: 1.25 - 3.48 Å).

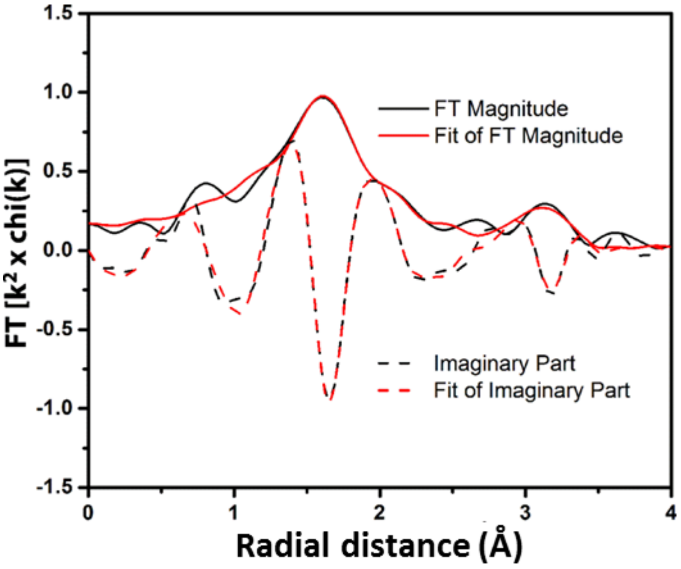

Scatterer	Zr-O_{coo}	Zr-$O_{\mu3\text{-}O}$	Zr···Zr
Coordination Number	1.9	4.0	2.1
Bond Distance (Å)	2.33	2.16	3.54
σ^2	0.003	0.004	0.010

Figure S8. Fitting results of the R-space EXAFS spectrum of UiO-66 after compression at 1.9 GPa (FT range: 3.5-12.5 $Å^{-1}$; fitting range: 1.25 - 3.48 Å).

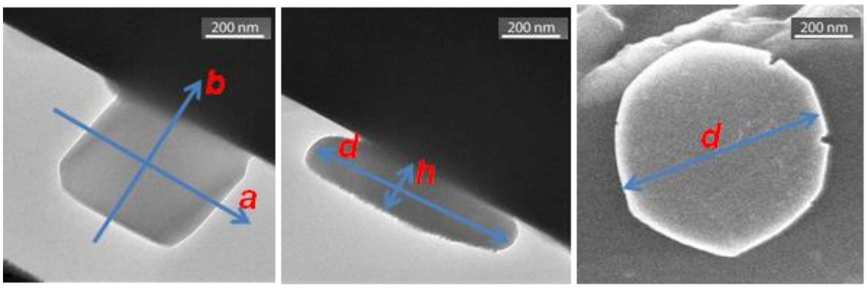

Figure S9. In situ TEM images taken before and after compression. Calculation of effective pressure and mass of single UiO-66 nanocrystal can be derived from these images**.** After loading UiO-66 on the silicon wedge holder, projection of the octahedral crystal of UiO-66 is diamond-shaped in the TEM. After application of pressure, the UiO-66 nanocrystal flattened along the compression direction (noted as b or h direction in TEMs above), and synchronously expanded along the parallel direction to the holder surface (a or d direction) and perpendicular to the direction of compression. Compressed UiO-66 nanocrystals resemble the shape of flattened pillar or pancake. We used a simple scheme to calculate the effective applied pressure: the diameter (d) of the contact surface and the height (h) between the flat punch and UiO-66 nanocrystal are measured from the *in-situ* video, where the loading and unloading force (F) were recorded from the pressure transducer used to apply the force. Thus, the cross-sectional contact surface (A) and the effective applied pressure (P) can be calculated as[2]

$$A = \pi\left(\frac{d}{2}\right)^2 \ (\mu m^2)$$

$$P = \frac{F}{A} = \frac{4F}{\pi d^2} \ (\text{GPa})$$

The mass of the individual UiO-66 nanocrystal (m) could be calculated considering the density of UiO-66 nanocrystal (ρ) of 1.238 g/ml,[3]

$$m = \rho V_0 = \rho h_0 A_0 = \rho h_0 \pi\left(\frac{d_0}{2}\right)^2$$

where V_0, h_0, d_0 are the initial volume, height and diameter of UiO-66 nanocrystal.

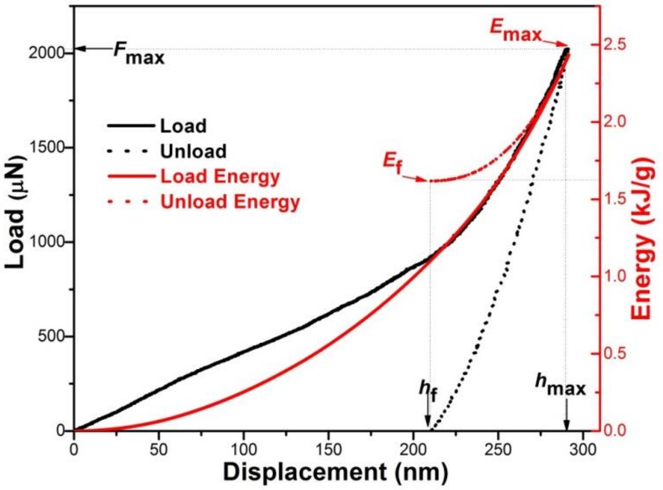

Figure S10. Calculation of the absorbed mechanical energy per gram during the loading and unloading process in the flat punch compression experiment. In general, the mechanical energy absorbed by the single UiO-66 nanocrystal during the loading and unloading processes can be expressed as

$$E = \int F dh = \int P dV$$

where E represents the mechanical energy absorbed (which we normalize to the mass of the individual nanocrystal), F and h are the load (μN) and displacement (nm), P and dV are the applied pressure (GPa) and the volume changes (μm^3), respectively. F and h can be directly read from the load-displacement curve. The flat punch uniaxially compresses the UiO-66 nanocrystal to the maximum load (F_{max}) and the maximum displacement (h_{max}), when the maximum amount of mechanical energy (E_{max}) is absorbed, followed by release of the pressure. In the unloading process, the UiO-66 nanocrystal recovers partially from an elastic deformation, rebounding partially to a final displacement (h_f). During the loading-unloading cycle, the absorbed mechanical energy per gram by a single UiO-66 nanocrystal from inelastic deformation is E_f (kJ/g).

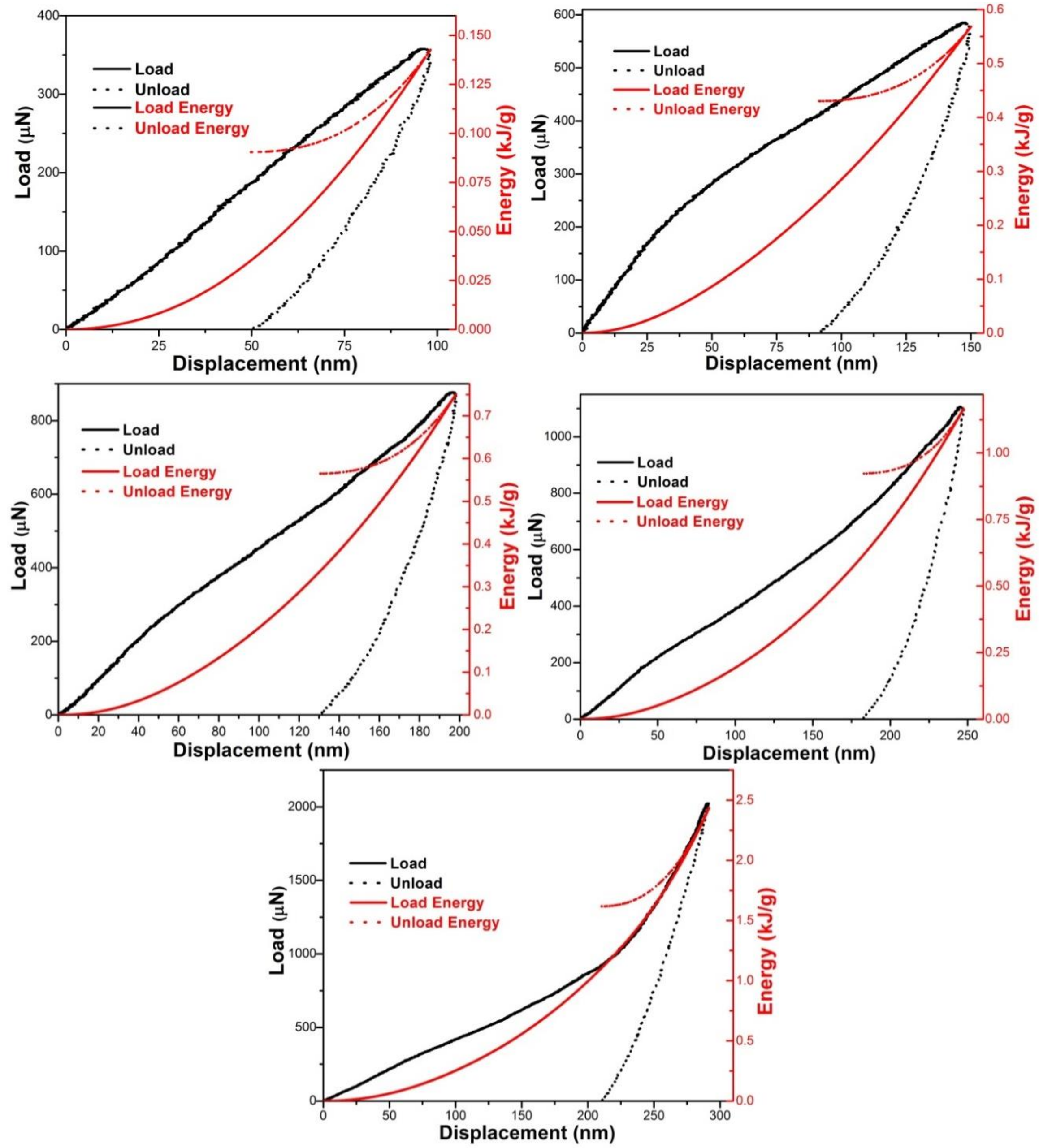

Figure S11. Representative load-displacement curves for individual UiO-66 nanocrystals (black) with variable maximum displacements around 100 nm, 150 nm, 200 nm, 250 nm and 300 nm, respectively. Right Y axis (red): The mechanical energy absorbed during compression by the UiO-66 nanocrystal. The solid and dotted lines are the experimentally observed loading and unloading processes, respectively; the absorbed mechanical energy so generated as a function of the maximum applied pressure is shown in Figure 6.

References

1. J. B. DeCoste, G. W. Peterson, B. J. Schindler, K. L. Killops, M. A. Browe and J. J. Mahle, *J. Mater. Chem. A*, 2013, **1**, 11922-11932.
2. Z. Su, Y.–R. Miao, S.–M. Mao, G. H. Zhang, S. Dillon, J. T. Miller, K. S. Suslick, *J. Am. Chem. Soc.* 2015, **137**, 1750-1753.
3. S. Øien, D. Wragg, H. Reinsch, S. Svelle, S. Bordiga, C. Lamberti, K. P. Lillerud, *Cryst. Growth Des.* 2014, **14**, 5370-5372.
